# Pharmacokinetics and tolerability of NSC23925b, a novel P-glycoprotein inhibitor: preclinical study in mice and rats

**DOI:** 10.1038/srep25659

**Published:** 2016-05-09

**Authors:** Yan Gao, Jacson K. Shen, Edwin Choy, Zhan Zhang, Henry J. Mankin, Francis J. Hornicek, Zhenfeng Duan

**Affiliations:** 1 Sarcoma Biology Laboratory, Center for Sarcoma and Connective Tissue Oncology, Massachusetts General Hospital and Harvard Medical School, Boston, MA 02114, USA; 2 Department of Clinical Laboratory Diagnostics, Beijing Friendship Hospital, Capital Medical University, Beijing 100050, China; 3 Shangqiu Medical College, Shangqiu 476100, Henan Province, China

## Abstract

Overexpression of P-glycoprotein (Pgp) increases multidrug resistance (MDR) in cancer, which greatly impedes satisfactory clinical treatment and outcomes of cancer patients. Due to unknown pharmacokinetics, the use of Pgp inhibitors to overcome MDR in the clinical setting remains elusive despite promising *in vitro* results. The purpose of our current preclinical study is to investigate the pharmacokinetics and tolerability of NSC23925b, a novel and potent P-glycoprotein inhibitor, in rodents. Plasma pharmacokinetic studies of single-dose NSC23925b alone or in combination with paclitaxel or doxorubicin were conducted in male BALB/c mice and Sprague-Dawley rats. Additionally, inhibition of human cytochrome P450 (CYP450) by NSC23925b was examined *in vitro*. Finally, the maximum tolerated dose (MTD) of NSC23925b was determined. NSC23925b displayed favorable pharmacokinetic profiles after intraperitoneal/intravenous (I.P./I.V.) injection alone or combined with chemotherapeutic drugs. The plasma pharmacokinetic characteristics of the chemotherapy drugs were not affected when co-administered with NSC23925b. All the animals tolerated the I.P./I.V. administration of NSC23925b. Moreover, the enzymatic activity of human CYP450 was not inhibited by NSC23925b. Our results demonstrated that Pgp inhibitor NSC23925b exhibits encouraging preclinical pharmacokinetic characteristics and limited toxicity *in vivo*. NSC23925b has the potential to treat cancer patients with MDR in the future.

P-glycoprotein (Pgp, MDR1, or ABCB1) has been identified as a pivotal modulator in a network of cellular factors that introduces multidrug resistance (MDR)^1^,^2^. The presence of Pgp overexpression in neoplasms is either inherent or acquired by the administration of chemotherapy. Increased expression of Pgp can induce MDR by preventing sufficient intracellular accumulation of a large number of anticancer drugs that are chemically, structurally, and functionally unrelated^3^. Initially, Pgp was recognized as merely responsible for the failure of conventional cytotoxic agents (e.g., vinblastine, doxorubicin, and paclitaxel). However, the actions of this transporter can contribute to resistance to a broad range of more than 300 compounds^4^,^5^. In addition, targeted therapy drugs are also substrates of this energy-dependent efflux pump^6^,^7^. For instance, constitutive overexpression of Pgp results in acquired resistance to the small molecule kinase inhibitor imatinib^8^. Therefore, suppression of Pgp expression and inhibition of its function have become promising strategies to overcome the development of MDR.

Ongoing efforts have focused on identifying more potent and specific compounds for the reversal of Pgp-mediated MDR^9^. These inhibitors can be produced by *de novo* synthesis using a variety of combinatorial chemistry approaches and a database of structural activity relationships for drug-Pgp interactions. A series of Pgp modulators or inhibitors have been demonstrated to be able to dramatically increase the chemosensitivity to common Pgp substrates in drug-resistant cell lines. Unfortunately, in spite of those promising *in vitro* results, almost none of the Pgp inhibitors have achieved clinical success over the years^3^,^10^,^11^. In general, these compounds, such as verapamil, cyclosporine A, valspodar (PSC833, Amdray), and biricodar (VX710, INCEL), were beset by poor potency, off-target effects, and toxicity^12^,^13^. A common strategy to overcome MDR is the co-administration of Pgp inhibitors with chemotherapy drugs. The failure to reverse MDR using Pgp inhibitors in clinic may also be attributed to adverse drug-drug interactions and unpredicted pharmacokinetic issues. For example, valspodar has shown undesirable pharmacokinetic behavior by interacting with paclitaxel, doxorubicin, etoposide, and mitoxantrone^14^,^15^. Additionally, similar pharmacokinetic profiles were observed in a potent Pgp inhibitor, tariquidar. Co-administration of tariquidar with vinorelbine demonstrated limited clinical activity^16^,^17^. Clearly, the clinical application of a new biologically active compound can be significantly constrained by its absorption, distribution, metabolism, excretion, and toxicity parameters within the body. Pharmacological studies are indispensable during Pgp inhibitor discovery and development.

NSC23925 has been identified as a potent MDR mitigator via selectively targeting Pgp by screening over 2000 small molecule compounds in the National Cancer Institute (NCI) Diversity Set library^18^,^19^. Four distinct isomers of NSC23925 exist as a result of two chiral centers of the structure, known as NSC23925a, NSC23925b, NSC23925c, and NSC23925d. Isomer NSC23925b shows the most potent bioactivities in reversing MDR. Previous studies have indicated that NSC23925b is able to reverse paclitaxel, doxorubicin, and mitoxantrone resistance in a human breast cancer MDR cell line and a human colon cancer MDR cell line. It is much more potent (10- to 60-fold) than that of the known drug resistance reversing agents verapamil or CsA^18^,^19^. Additionally, NSC23925b is also able to prevent the emergence of anticancer drug resistance *in vivo* and *in vitro* by suppressing Pgp function, as shown in ovarian cancer and osteosarcoma^20^,^21^. Even though NSC23925b holds therapeutic value in the treatment of MDR-dependent cancers, its pharmacokinetic behavior is largely unknown. Currently, there are no pharmacokinetic or toxicity data for NSC23925b.

The purposes of the present study are to characterize the *in vivo* pharmacokinetics of isomer NSC23925b in rodents, to evaluate *in vitro* human Cytochrome P450 (CYP450) inhibitory properties, and to investigate the preclinical maximum tolerated dose and safety profile of this small molecular compound (Relative molecular mass *M*_r_ = 421.35).

## Results

### Plasma pharmacokinetic profile of NSC23925b after I.P./I.V. injection alone or combined with chemotherapeutic drug

**Figure 1:**
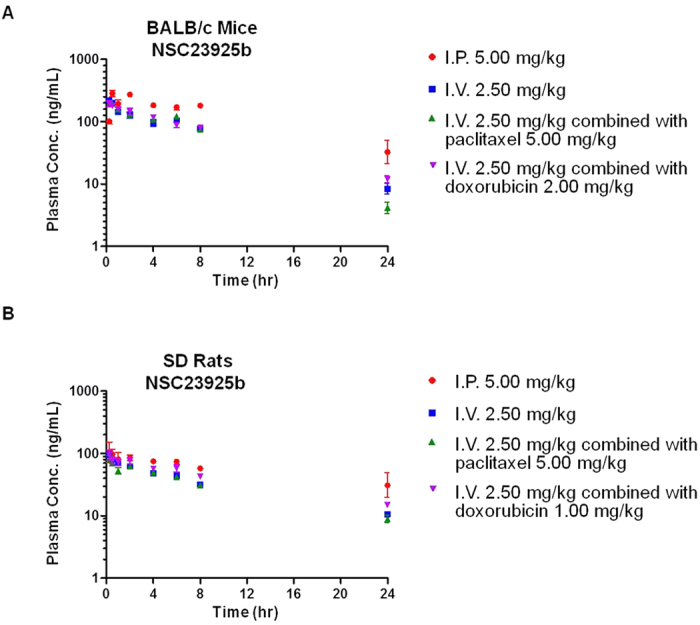
Plasma pharmacokinetic profile of NSC23925b after intraperitoneal injection/intravenous injection (I.P./I.V.) administration. (**A**) Plasma concentration-time profile of NSC23925b in male BALB/c mice at 5.00 mg/kg (I.P.) alone, 2.50 mg/kg (I.V.) alone, and co-administered with paclitaxel/doxorubicin; (**B**) single-dose plasma pharmacokinetics of NSC23925b in male Sprague-Dawley rats following 5.00 mg/kg (I.P.) alone, 2.50 mg/kg (I.V.) alone, and co-administered with paclitaxel/doxorubicin. Data: mean ± SD of each time point.

The mean plasma concentration versus time profiles of NSC23925b (I.P.: 5.00 mg/kg, I.V.: 2.50 mg/kg) when administered alone or with paclitaxel/doxorubicin are shown in Fig. 1, and the corresponding pharmacokinetic parameters are summarized in Tables 1 and S1. In brief, peak concentrations were achieved at approximately 1.50 ± 0.866 hours (mice) and 2.17 ± 3.32 hours (rats) post-I.P. dose of NSC23925b. NSC23925b concentrations declined biexponentially after the T_max_ with a mean terminal elimination half-life of 8.69 ± 4.18 hours for mice and 9.24 ± 3.77 hours for rats. Following I.V. administration to mice, mean clearances (CL) were 24.6 ± 1.96 mL·kg^−1^·min^−1^, 25.3 ± 0.984 mL·kg^−1^·min^−1^, and 22.8 ± 0.938 mL·kg^−1^·min^−1^ for NSC23925b alone, NSC23925b with paclitaxel, and NSC23925b with doxorubicin, respectively. There were no significant changes among other pharmacokinetic parameters; for example, in the case of AUC_0-inf_ (the area under the plasma concentration versus time curve), NSC23925b only was 1701 ± 130 h·ng·mL^−1^, combination NSC23925b and paclitaxel was 1649 ± 65.6 h·ng·mL^−1^, combination NSC23925b and doxorubicin and 1827 ± 73.8 h·ng·mL^−1^. The peak plasma NSC23925b concentrations for both I.V. and I.P. were observed at the earliest time point (15 minutes after administration). The concentrations of NSC23925b in plasma were above the lower limit of quantification throughout the sampling period. Similar to the results obtained in mice, the three pharmacokinetic profiles of NSC23925 alone and co-administered with either paclitaxel or doxorubicin also displayed a parallel trend within the rats.

### The pharmacokinetic characteristics of chemotherapeutic drugs (paclitaxel, doxorubicin) were not influenced when co-administrated with NSC23925b

**Figure 2:**
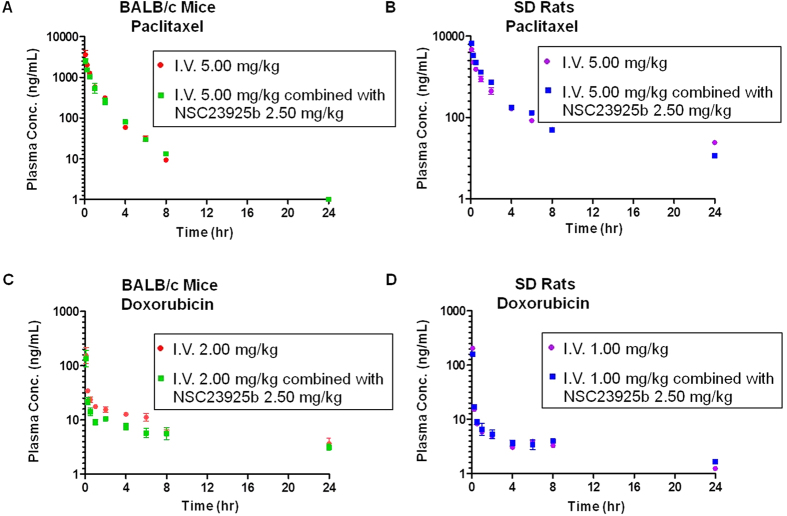
Plasma pharmacokinetic profile of paclitaxel and doxorubicin after intravenous injection (I.V.) alone or co-administration with NSC23925b. Plasma concentration-time course of paclitaxel in male BALB/c mice at a single 5.00 mg/kg dose (**A**), and in male Sprague-Dawley rats at a single 5.00 mg/kg dose (**B**); plasma concentration-time curves of doxorubicin in male BALB/c mice at a single 2.00 mg/kg dose (**C**), and in male Sprague-Dawley rats at a single 1.00 mg/kg dose (**D**). Data: mean ± SD of each time point.

The mean plasma concentration versus time profiles of paclitaxel and doxorubicin during I.V. administration combined with or without NSC23925b are shown in Fig. 2. The overall plasma concentration-time profiles for the two chemotherapeutic drugs demonstrate a rapid decline in concentrations. Moreover, for either paclitaxel or doxorubicin, as well as either mice or rats, when the drugs were used alone or in combination with NSC23925b, these curves almost overlapped. The corresponding pharmacokinetic parameters are summarized in Tables 2 and S2. Paclitaxel concentrations in mice displayed a mean terminal elimination half-life of 1.33 ± 0.28 hours when dosed alone, and 1.54 ± 0.115 hours while dosed together with NSC23925b. Doxorubicin concentrations in mice showed a similar mean terminal elimination half-life, 10.7 ± 3.29 hours (dosed alone) vs. 12.8 ± 1.91 hours (dosed together with NSC23925b). With respect to the CL of paclitaxel and doxorubicin, similar values were obtained: 33.2 ± 3.12 mL·kg^−1^·min^−1^ (dosed alone) compared with 39.8 ± 6.46 mL·kg^−1^·min^−1^ (dosed together with NSC23925b) for paclitaxel, and 18.2 ± 1.93 mL·kg^−1^·min^−1^ (dosed alone) compared with 14.6 ± 1.53 mL·kg^−1^·min^−1^ (dosed together with NSC23925b) for doxorubicin. However, some of the pharmacokinetic parameters of chemotherapeutic drug paclitaxel, such as Vd_ss_, t_1/2_, K_el_, MRT_IV_, and AUMC_0-inf_ were altered when combined with NSC23925b in rats. Vd_ss_ can change independent of clearance. As a result, a decrease of Vd_ss_ in the absence of alteration in clearance resulted in shorter t_1/2_ and larger K_el_.

### NSC23925b did not inhibit the activity of human CYP450

**Figure 3:**
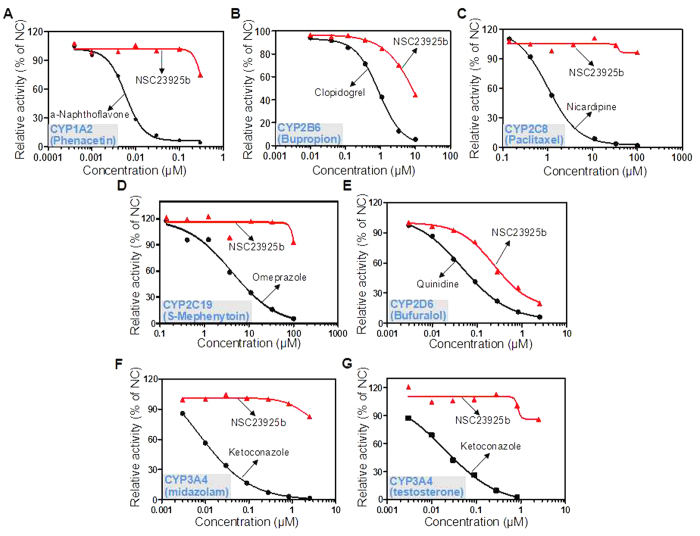
Comparison of inhibitive potentials between NSC23925b and reference inhibitors on human cytochrome (CYP) 450s (CYP1A2, 2B6, 2C8, 2C19, 2D6, and 3A4). The concentration-effect sigmoid-shaped plots of NSC23925b and particular reference inhibitor against the respective CYP450 enzyme, CYP1A2 (**A**), CYP2B6 (**B**), CYP2C8 (**C**), CYP2C19 (**D**), CYP2D6 (**E**), and CYP3A4 (**F**,**G**). The red triangles and line represent NSC23925b, and the black dots and line indicates the reference inhibitors. “NC” stands for normal control (saline).

*In vitro* CYP450 inhibition evaluation of NSC23925b was conducted in the HLM reaction system for the CYP1A2, 2B6, 2C8, 2C9, 2C19, 2D6, and 3A4 enzymes, respectively. Thirty μM phenacetin (substrate of CYP1A2), 70 μM bupropion (substrate of CYP2B6), 10 μM paclitaxel (substrate of CYP2C8), 10 μM diclofenac (substrate of CYP2C9), 35 μM S-mephenytoin (substrate of CYP2C19), 10 μM bufuralol (substrate of CYP2D6), 5 μM midazolam, and 80 μM testosterone (substrates of CYP3A4) were treated with HLM in the presence of their specific inhibitors or NSC23925b. The substrate-CYP450 enzyme reactions and reference inhibitors are shown in Table 3. A sigmoid-shaped curve (Log(inhibitor) vs. Response-Variable slope) was fitted to the data and the IC_50_ was calculated using GraphPad Prism software. The concentration-effect plots of NSC23925b and particular reference inhibitor against the CYP450 enzyme are displayed in Fig. 3. As expected, specific CYP inhibitors showed significant inhibitory activity after incubating with the substrates (Table 3). Although NSC23925b was found to be a moderate inhibitor on CYP2B6 and CYP2D6 mediated metabolism of bupropion and bufuralol, respectively, the IC_50_ values of NSC23925b were still much higher than the reference inhibitors clopidogrel and quinidine (8.589 versus 0.914, 1.407 versus 0.048). Moreover, the IC_50_s of NSC23925b on CYP450 mediated metabolism of other standard substrates were found to be >10 μM.

### The I.P./I.V. administration of NSC23925b was well-tolerated by BALB/c mice and SD rats

During the pharmacokinetic study, I.P. or I.V. administrations of NSC23925b with or without paclitaxel/doxorubicin were generally tolerated by all the rodents. Specifically, all 180 mice and 24 rats were administered the aforementioned intended doses; no treatment-related clinical signs were observed from the animals. BALB/c mice had body weights ranging from 17.80 to 22.80 g; SD rats had body weights ranging from 225 to 255 g. A total of 80 (40 female, 40 male) mice and 80 (40 female, 40 male) rats were used in the sighting study and main study on assessing the MTD of NSC23925b. For I.P. injection, three of five female mice survived after administering 93.75 mg/kg NSC23925b, while all five male mice tolerated the dosage of 68.80 mg/kg; one of five female rats died when treated with 47.20 mg/kg, and all male rats were still in good status at 33.10 mg/kg dose (Table 4). In regard to the I.V. dosing route, the 24.70 mg/kg dosage did not significantly influence the health conditions of any of the female and male mice, while female and male rats generally tolerated as much as 47.20 mg/kg and 38.80 mg/kg NSC23925b respectively, with the exception of one female rat that was moribund (Table 4).

## Discussion

NSC23925b is a novel compound that reverses and prevents Pgp-mediated MDR in cancer cells^18^,^19^,^22^,^23^,^24^. In the current study, we have determined the plasma pharmacokinetics of NSC23925b after I.P or I.V administration in mice and rats. Following the I.P. 5.00 mg/kg dose and the I.V. administration of a single 2.50 mg/kg dose of NSC23925b, the highest concentrations in plasma were observed at approximately 1–2 hours after I.P. injection and 5 min after I.V. injection. Rapid decline in concentration of NSC23925b was followed by a steady elimination in mice and rats. NSC23925b also showed high volumes of distribution at a steady state (>9 L/kg). The demonstrated concentration-time profiles were analogous to some reported drug resistance inhibitors^25^,^26^,^27^. The peak plasma inhibitor concentrations were observed at the earliest time point especially after I.V. injection, and the concentrations of detected inhibitor in plasma were above the lower limit of quantification throughout the sampling period. In addition, the pharmacokinetics of classic chemotherapeutic drugs, paclitaxel and doxorubicin, were not affected when combined with NSC23925b. Although it seems that some pharmacokinetic parameters, such as K_el_, t_1/2_, MRT_IV_, and Vd_ss_, of the chemotherapeutic drug paclitaxel were altered when combined with NSC23925b in rats, these results may be explained as changes in volume of distribution were independent of changes in clearance^28^. Further study of pharmacokinetics and NSC23925b in large animals, such as in dogs or monkeys, may be needed to validate these changes. Moreover, whether NSC23925b could be extrapolated to restore targeted therapy drugs sensitivity requires further investigation.

Human CYP450 is a family of enzymes responsible for the essential biotransformation processes of endogenous molecules and xenobiotics^29^. Alteration of CYP450 enzymatic activity, such as inhibition or induction, can remarkably impact the pharmacokinetics and thereby the pharmacodynamics of drugs. Particularly, enzyme inhibition by a specific drug is the main cause of adverse side-effects^22^,^30^. For that purpose, CYP450 inhibition data are useful in designing strategies for investigating clinical drug-drug interaction studies. The IC_50_ values of drugs, which represent the concentration of inhibitor required for 50% inhibition of enzyme activity, are determined in order to estimate the inhibition potential toward all corresponding CYPs. IC_50_ values of >10 μM, 1–10 μM, and <1 μM are considered weak, moderate, and potent inhibitors, respectively^31^. Based on the *in vitro* NSC23925b CYP450 inhibition assay results, the IC_50_s of this compound for human CYP450 enzymes (CYP1A2, 2C8, 2C9, 2C19, and 3A4) were more than 10 μM and could be regarded as a weak inhibitor. In addition, NSC23925b was considered as moderate inhibitor for CYP2B6 and CYP2D6 (the IC_50_s were between 1–10 μM). Even though the IC_50_ of NSC23925b for CYP2D6 was 1.407 μM, it was still approximately 30 times higher than the relative reference inhibitor (0.048 μM). Moreover, co-administration may result in inhibition of the metabolism of one or both compounds through inhibition of a specific CYP450 enzyme. NSC23925b has again been confirmed to not act as an inhibitor of any CYP450 enzymes due to its weak influence on the plasma pharmacokinetic characters of paclitaxel/doxorubicin in the abovementioned study. Therefore, it may be deduced that NSC23925b will not alter pharmacokinetics, distribution, or clearance of the commonly used chemotherapeutic drugs.

In general, all treatments were well-tolerated by the animals across the pharmacokinetic study and the MTD assay. Adverse events were generally mild, either from the known effects of the chemotherapeutic drugs studied or other unrelated toxic reactions, and were resolved without intervention. Particularly, critical negative consequences of paclitaxel include neurotoxicity, hypersensitivity, hematologic toxicity, cardiac disturbances, and gastrointestinal tract symptoms, while the major side-effect of doxorubicin is cardiotoxicity, which can lead to cardiomyopathy and congestive heart failure^23^,^24^. However, during the pharmacokinetic study, the combination of NSC23925b did not aggravate the adverse reactions of the two drugs in their *in vivo* safe dose range. Moreover, according to the MTD results, the rodents tolerated the dosage of NSC23925b from 24.70 mg/kg to 93.75 mg/kg, which was much higher than the 2.50–5.00 mg/kg dose used in previous pharmacokinetic analyses and investigations^18^,^20^,^32^. Hence, a wide range of dosages of NSC23925b can be safely utilized in preclinical studies, which may offer a hopeful perspective for its application in Phase I/II clinical study.

In conclusion, NSC23925b is a potential Pgp inhibitor with favorable preclinical pharmacokinetics and limited toxicity. The data in the current study will provide insights on Phase I/II clinical trials evaluating NSC23925b alone or combined with chemotherapeutics. Moreover, we have crystallized isomers of NSC23925b and collected the crystal structure data. These results will also facilitate the initiation of an ongoing docking model to understand the underlying mechanism of how NSC23925b inhibits the function of Pgp.

## Methods

### Drug and chemical information

The NSC23925b isomer (molecular weight: 384.9 g/mol) was synthesized by Chengdu ChemPartner Co., Ltd. with a purity of >99%. NSC23925b was initially dissolved in 5% dimethyl sulfoxide (DMSO), and then diluted in normal saline to the concentration as required. Final vehicle concentrations were 1–5% DMSO and 95–99% saline. Paclitaxel (6 mg/mL) and doxorubicin (2 mg/mL) were supplied by Teva Pharmaceuticals (Sellersville, PA) and APP Pharmaceuticals (Schaumburg, IL), respectively. Both chemotherapeutic drugs were diluted with normal saline for injection. DMSO was purchased from Sigma-Aldrich (St. Louis, MO). Clinical use saline was obtained from Jiling Dubang Pharmaceutical Co., Ltd. The dose levels of NSC23925b, paclitaxel, and doxorubicin were determined based on available data from previous related studies^18^,^19^,^20^,^21^. The particular dosage of each chemical is described in the following specific experiments. All other reagents utilized in this study were provided by PharmaLegacy Laboratories (Shanghai, China). Data on handling and disposal instructions together with any available safety information are retained on file at PharmaLegacy Laboratories.

### Animals

Naive BALB/c mice and Sprague-Dawley rats (SD rats) were ordered from Shanghai SLAC Laboratory Animal Co. Ltd (Shanghai, China). All the animals were specific pathogen free and approximately 6–8 weeks old when they arrived at PharmaLegacy Laboratories. The mice were 18–22 g, and the rats were 200–250 g upon receipt. A health inspection was performed on each animal to include evaluation of the coat, extremities, and orifices. Each animal was also examined for any abnormal signs in posture or movement. The animals were housed in the PharmaLegacy Laboratories vivarium in clear polycarbonate plastic cages. The adaptation to the environment for the animals was no less than seven days. Animals were observed daily for signs of ill health and general reaction to the treatments. The procedures that were applied on animals in this study have been approved by the Massachusetts General Hospital Subcommittee on Research Animal Care (protocol number 2013N000121) and PharmaLegacy Laboratories Institutional Animal Care and Use Committee. The methods were carried out in accordance with the approved guidelines. Any moribund animals were euthanized with CO_2_ inhalation followed by cervical dislocation for humane reasons. All surviving animals placed on study were euthanized at their scheduled study termination. Extra animals obtained for this study, but not placed on study, were returned to PharmaLegacy vivarium for other uses, such as training programs.

### *In vivo* preclinical pharmacokinetic analyses

Pharmacokinetic studies of NSC23925b, paclitaxel, and doxorubicin were performed in male BALB/c mice and SD rats. Specifically, 5.00 mg/kg (dose concentration: 0.50 mg/mL) and 2.50 mg/kg (dose concentration: 0.25 mg/mL) NSC23925b were administered by intraperitoneal and intravenous (I.P. and I.V.) injection, respectively; 5.00 mg/kg (dose concentration: 0.50 mg/mL) paclitaxel was administered to all murine via I.V.; and 2.00 mg/kg (dose concentration: 0.20 mg/mL) doxorubicin for mice and 1.00 mg/kg (dose concentration: 0.10 mg/mL) doxorubicin for rats were administered through I.V.

After dosing, blood samples were collected at different time points for each group. The time points for the I.P. dosing groups were at pre-dose and post dose (15, 30 min, and then 1, 2, 4, 6, 8, and 24 hours) of the test chemicals; for the I.V. dosing groups, time points were at pre-dose and post dose (5, 15, 30 min, and then 1, 2, 4, 6, 8 and 24 hours) of the test agents. Three animals were used for each time point. Approximately 800 μL of blood was collected via orbital vein (a source of collecting sufficient volume of venous blood) from each anesthetized mouse by isoflurane, and 200 μL of blood was collected from the cannula implanted in the jugular vein. The catheter was implanted when the blood sample was collected at the first time point. Next, the blood was transferred to a prepared tube containing sodium heparin anticoagulant and mixed briefly by gentle inversion three to four times. The target and actual time of blood sampling were recorded relative to the time of dosing. Samples were placed on wet ice immediately after collection. Within 1 hour after collection, plasma was separated by centrifugation at 4,000 rpm for 10 minutes at 4 °C. The plasma was protected from light and stored at −80 °C until analyzed by liquid chromatography-mass spectrometry/mass spectrometry (LC-MS/MS) for quantification.

Standards were prepared in blank murine plasma that was in a tube containing sodium heparin anticoagulant covering the nominal concentration range of 2–1,000 ng/mL (NSC23925b), 2.50–1,000 ng/mL (paclitaxel), and 1–1,000 ng/mL (doxorubicin). Using the data from the standards, calibration curves were generated for each analytical batch. Aliquots of 10.0 μL of samples, calibraton curve samples, and QC samples were supplemented with 100 μL of precipitant (a mixture of acetone and acetonitrile (30:70)). After vortexing for 3 min and centrifuging at 12,000 rpm for 3 min, 75.00 μL of the supernatant was transferred into a 96-well microplate with an equal volume of water. The 10 μL mixture solution was injected for LC-MS/MS analysis. The specific instrumental conditions of LC-MS/MS bioanalysis of NSC23925b, paclitaxel, and doxorubicin are listed in Table 5. The estimation of pharmacokinetic parameters is shown in Tables 1, 2, S1 and S2.

### *In vitro* human liver microsomes (HLM)-LC-MS human CYP450 inhibition study of NSC23925b

To understand the potential drug-drug interactions of NSC23925b, the HLM-LC-MS CYP450 inhibition assay was performed *in vitro*. This assay used a cocktail of drug-probe substrates that are specific to each isozyme with HLM and LC-MS detection^33^,^34^. Each test article and reference inhibitor was dissolved in 40% DMSO and 60% acetonitrile (ACN) mixture. Specifically, 0.20 mg/mL HLM, test compound set, and reference inhibitor solution were added into the designated 96-well polypropylene plate. Substrates were then added to their respective well containing the compound/HLM solution to start the reaction. The drug-probe substrate concentrations and estimated individual CYP enzyme concentrations from HLM are shown in Table 3. Nicotinamide adenine dinucleotide phosphate (NADPH) cofactor (66.70 mg NADPH in 10 mL 100 mM phosphate buffer, pH 7.4) was added into each well. After incubating for 5 min for 3A4; 10 min for 1A2, 2C8, 2C9, and 2D6; and 45 min for 2C19 at a 37 °C incubator, the reactions were terminated by addition of 120 μL of ACN to denature the protein. The solution was centrifuged at 4 °C for 30 min at 3,700 rpm and the supernatant was transferred for LC-MS analysis. The concentrations of the metabolites were determined from standard curves using commercially available metabolite standards. Compounds were tested in triplicate. The half maximal inhibitory concentration (IC_50_) values were determined by plotting the metabolite formation over the logarithm of the compound concentration using Prism 5.0 software (GraphPad Software Inc., San Diego, CA).

### Determination of maximum tolerated dose (MTD) of NSC23925b and evaluation of acute safety profiles in rodents

The MTD determination assay was composed of sighting study and main study^26^,^35^,^36^,^37^. For the sighting study of the determination maximum tolerable dose (MTD) of NSC23925b, one of each animal (one female rat, one male rat, one female mouse, and one male mouse) was used at each I.V. and I.P. injection dose level. The starting dose was selected from the fixed dose levels (5, 50, 500, 2000 mg/kg) as a dose expected to produce evident toxicity. The first animal was dosed and observed. Time of appearance, degree, and lasting/recovery time of signs were recorded in details. Afterwards, animals were observed twice on the dosing day and then changed to daily observation. The observation lasted at least 14 days. If the first animal died or appeared impaired, the second animal received a middle dose of the two doses and vice versa. The sighting study was completed when a decision on the starting dose for the main study was made.

During the main study, a total of five animals of one sex were used for each dose level investigated. The five animals were made up of one animal from the sighting study dosed at the selected dose level together with an additional four animals. Each group received one of the fixed doses by I.V. and I.P. based on the sighting study. If the first animal died or appeared impaired, the second animal received a middle dose of the two doses and vice versa.

Animals were fasted overnight prior to dosing. Following the period of fasting, the animals were weighed and the dose was calculated according to the body weight. After the animals were administrated a dose volume of 10 mL/kg, food was withheld for another 3–4 hours. Individual weights of animals were determined shortly before the test substance was administered and twice weekly thereafter. Weight changes were calculated and recorded. Animals injected with saline were the control group for the weight alteration.

### Data collection and statistical methods

Results were expressed as mean ± SD and 95% confidence interval. Data collection was performed using Analyst version 1.4 from Applied Biosystems. All pharmacokinetic parameters of NSC23925b, paclitaxel, and doxorubicin after I.V. or I.P. administrations in mice/rats were calculated based on the plasma concentrations obtained in this study by non-compartmental analysis by WinNonLin software (Version 5.01, Pharsight, Inc., Mountain View, CA). The data were also analyzed using Prism 5.0 software. Statistical significance was determined using independent two-tailed Student t-tests and one-way ANOVA for independent data. Differences of *P* < 0.05 were regarded as significant for all statistical analyses.

## Additional Information

**How to cite this article**: Gao, Y. *et al*. Pharmacokinetics and tolerability of NSC23925b, a novel P-glycoprotein inhibitor: preclinical study in mice and rats. *Sci. Rep*. **6**, 25659; doi: 10.1038/srep25659 (2016).

## Supplementary Material

Supplementary Tables

## Figures and Tables

**Table 1:** Plasma pharmacokinetic parameters of NSC23925b (Data provided as mean ± SD).

Pharmacokinetic Parameters	BALB/c Mice				SD Rats			
	I.P. 5.00 mg/kg	I.V. 2.50 mg/kg	I.V. 2.50 mg/kg		I.P. 5.00 mg/kg	I.V. 2.50 mg/kg	I.V. 2.50 mg/kg	
			Combined with Paclitaxel 5.00 mg/kg	Combined with Doxorubicin 2.00 mg/kg			Combined with Paclitaxel 5.00 mg/kg	Combined with Doxorubicin 1.00 mg/kg
K_el_(h^−1^)	0.0922 ± 0.0402	0.140 ± 0.0172	0.185 ± 0.0281	0.114 ± 0.0199	0.0826 ± 0.0280	0.0785 ± 0.00486	0.0842 ± 0.0102	0.0711 ± 0.00389
t_1/2_(h)*	8.69 ± 4.18	5.01 ± 0.661	3.81 ± 0.569	6.17 ± 0.985	9.24 ± 3.77	8.86 ± 0.541	8.32 ± 1.07	9.77 ± 0.535
AUC_0-t_ (h·ng·mL^−1^)	3364 ± 197	1636 ± 114	1624 ± 75.9	1716 ± 101	1386 ± 173	766 ± 51.9	725 ± 49.0	979 ± 109
AUC_0-inf_ (h·ng·mL^−1^)	3983 ± 819	1701 ± 130	1649 ± 65.6	1827 ± 73.8	1994 ± 855	902 ± 57.4	837 ± 77.1	1191 ± 133
AUMC_0-t_ (h·h·ng·mL^−1^)	25329 ± 4810	9893 ± 1299	9122 ± 493	10730 ± 314	13328 ± 5611	5504 ± 317	5110 ± 574	7447 ± 855
AUMC_0-inf_ (h·h·ng·mL^−1^)	50744 ± 36083	11945 ± 2141	9862 ± 253	14400 ± 1089	38450 ± 36415	10502 ± 642	9163 ± 2323	15529 ± 2132
CL (mL·kg^−1^·min^−1^)	/	24.6 ± 1.96	25.3 ± 0.984	22.8 ± 0.938	/	46.3 ± 3.06	50.1 ± 4.75	35.3 ± 4.11
MRT_IV_(h)	/	6.99 ± 0.836	5.98 ± 0.166	7.90 ± 0.788	/	11.7 ± 0.512	10.9 ± 1.89	13.0 ± 0.964
Vd_SS_(L·kg^−1^)	/	10.3 ± 0.808	9.09 ± 0.565	10.8 ± 1.44	/	32.4 ± 3.06	32.4 ± 3.87	27.6 ± 3.42
t_max_(h)	1.50 ± 0.866	/	/	/	2.17 ± 3.32	/	/	/
C_max_(ng·mL^−1^)	298 ± 56.1	/	/	/	128 ± 44.3	/	/	/
F (%)	117.1 ± 24.06	/	/	/	110.6 ± 47.4	/	/	/

Abbreviations: AUC_0-t_, area under the concentration-time curve calculated from zero up to the last measured concentration; AUC_0-inf_, area under the concentration-time curve extrapolated from zero up to infinity; AUMC_0-t_, area under the first moment of the concentration-time curve from zero up to the last measured concentration; AUMC_0-inf_, area under the first moment of the concentration-time curve from zero up to infinity; CL, clearance; C_max_, maximum plasma concentration; F, bioavailability; MRT_IV_, mean residence time of a drug administered intravenously; I.P., intraperitoneal injection; I.V., intravenous injection; K_el_, elimination rate constant; t_max_, time to maximum observed concentration; t_1/2_, elimination half life; Vdss, volume of distribution at steady-state. *P < 0.05.

**Table 2:** Plasma pharmacokinetic parameters of paclitaxel and doxorubicin (Data provided as mean ± SD).

Pharmacokinetic Parameters	Paclitaxel				Doxorubicin			
	BALB/c Mice		SD Rats		BALB/c Mice		SD Rats	
	I.V. 5.00 mg/kg	Combined with NSC23925b 2.50 mg/kg	I.V. 5.00 mg/kg	Combined with NSC23925b 2.50 mg/kg	I.V. 2.00 mg/kg	Combined with NSC23925b 2.50 mg/kg	I.V. 1.00 mg/kg	Combined with NSC23925b 2.50 mg/kg
K_el_(h^−1^)	0.535 ± 0.102	0.453 ± 0.0343	0.0602 ± 0.00420	0.129 ± 0.00797†	0.0682 ± 0.0184	0.0548 ± 0.00810	0.0695 ± 0.0214	0.0486 ± 0.00290
t_1/2_ (h)	1.33 ± 0.28	1.54 ± 0.115	11.6 ± 0.777	5.40 ± 0.346†	10.7 ± 3.29	12.8 ± 1.91	10.5 ± 2.76	14.3 ± 0.870
AUC_0-t_ (h·ng·mL^−1^)	2504 ± 238	2104 ± 386	4200 ± 492	5677 ± 624	207 ± 9.76	158 ± 29.3	89.7 ± 35.6	105 ± 5.16
AUC_0-inf_ (h·ng·mL^−1^)	2523 ± 244	2134 ± 381	4608 ± 517	5767 ± 609	272 ± 53.7	218 ± 28.5	117 ± 32.5	139 ± 8.44
AUMC_0-t_ (h·h·ng·mL^−1^)	2728 ± 356	2642 ± 484	13231 ± 1265	12697 ± 484	1470 ± 309	1217 ± 180	446 ± 282	706 ± 51.6
AUMC_0-inf_(h·h·ng·mL^−1^)	2911 ± 400	2947 ± 425	29892 ± 4245	15585 ± 149†	4157 ± 2493	3766 ± 987	1318 ± 527	2233 ± 231
CL (mL·kg^−1^·min^−1^)	33.2 ± 3.12	39.8 ± 6.46	18.2 ± 1.93	14.6 ± 1.53	126 ± 22.4	155 ± 19.2	151 ± 43.1	120 ± 7.57
MRT_IV_ (h)	1.16 ± 0.147	1.39 ± 0.0829	6.50 ± 0.853	2.72 ± 0.304†	14.6 ± 5.71	17.3 ± 4.17	11.1 ± 2.41	16.1 ± 0.771
Vd_SS_(L·kg^−1^)	2.31 ± 0.428	3.33 ± 0.680	7.13 ± 1.34	2.40 ± 0.515†	105 ± 19.8	161 ± 47.9	97.1 ± 19.5	116 ± 3.57

Abbreviations: AUC_0-t_, area under the concentration-time curve calculated from zero up to the last measured concentration; AUC_0-inf_, area under the concentration-time curve extrapolated from zero up to infinity; AUMC_0-t_, area under the first moment of the concentration-time curve from zero up to the last measured concentration; AUMC_0-inf_, area under the first moment of the concentration-time curve from zero up to infinity; CL, clearance; MRT_IV_, mean residence time of a drug administered intravenously; I.P., intraperitoneal injection; I.V., intravenous injection; K_el_, elimination rate constant; t_1/2_, elimination half life; Vdss, volume of distribution at steady-state. ^†^P < 0.05.

**Table 3:** Comparison of inhibitive potentials IC_50_ between NSC23925b and reference inhibitors on cytochrome P450s (CYP1A2, 2B6, 2C8, 2C9, 2C19, 2D6, 3A4).

Cytochrome P450Isoforms	Substrate/ Concentration (μM)	Substrate Reaction	Compound IC_50_ (μM)	
			NSC23925b	Reference Inhibitors
CYP1A2	Phenacetin/30	*O*-deethylation	>10	0.006 a-Naphthoflavone
CYP2B6	Bupropion/70	hydroxylation	8.589	0.914 Clopidogrel
CYP2C8	Paclitaxel/10	6α-hydroxylation	>10	1.248 Nicardipine
CYP2C9	Diclofenac/10	4-hydroxylation	>10	0.635 Sulfaphenazole
CYP2C19	S-Mephenytoin/35	4-hydroxylation	>10	4.081 Omeprazole
CYP2D6	Bufuralol/10	*O*-demethylation	1.407	0.048 Quinidine
CYP3A4	Midazolam/5	1-hydroxylation	>10	0.015 Ketoconazole
	Testosterone/80	6ß-hydroxylation	>10	0.022 Ketoconazole

**Table 4:** Determination of maximum tolerated dose (MTD) of NSC23925b in rodents.

Animals	Route	Dose	Number of Death	Number of Exposure
BABL/c Mice	I.P.	93.75 mg/kg (Female)	2	5
	I.P.	68.80 mg/kg (Male)	0	5
	I.V.	24.70 mg/kg (Female)	0	5
	I.V.	24.70 mg/kg (Male)	0	5
SD Rats	I.P.	47.20 mg/kg (Female)	1	5
	I.P.	33.10 mg/kg (Male)	0	5
	I.V.	47.20 mg/kg (Female)	1	5
	I.V.	38.80 mg/kg (Male)	0	5

Abbreviations: I.P., intraperitoneal injection; I.V., intravenous injection.

**Table 5:** Instrumental conditions of LC-MS/MS bioanalysis of NSC23925b, paclitaxel and doxorubicin.

	NSC23925b	Paclitaxel	Doxorubicin
Column	Sepax GP-C18(2.1 × 50 mm, 3 μm);	AgelaVenusil XBP C18(L)(2.1 × 50 mm, 5 μm)	Sepax GP-C18(2.1 × 50 mm, 3 μm)
Mobile Phase	A: H_2_O (0.1% Formic acid);B: ACN (0.1% Formic acid);	A: H_2_O (0.1% Formic acid);B: ACN (0.1% Formic acid)	A: H_2_O (0.1% Formic acid);B: ACN (0.1% Formic acid)
Gradient	0.10 min: 90%A,10%B;1.00 min, 10%A, 90%B;2.00 min, 10%A, 90%B;2.01 min, 90%A, 10%B;3.00 min, 90%A, 10%B;	0.10 min: 90%A,10%B;0.60 min, 5%A, 95%B;1.60 min, 5%A, 95%B;1.61 min, 90%A, 10%B;3.00 min, 90%A, 10%B	0.10 min: 90%A,10%B;1.00 min, 10%A, 90%B;2.00 min, 10%A, 90%B;2.01 min, 90%A, 10%B;3.00 min, 90%A, 10%B
Flow Rate	0.30 mL/min	0.35 mL/min	0.30 mL/min
Mass Mode	ESI positive	ESI positive	ESI positive
Mass Transition	349.2 ≥ 266.2	854.5 ≥ 286.3	544.4 ≥ 397.1
Retention Time	1.95 min	1.72 min	1.93 min
