# Supplementary material for: Pharmacokinetics and tolerability of NSC23925b, a novel P-glycoprotein inhibitor: preclinical study in mice and rats
Source: Sci Rep. 2016 May 9;6:25659. doi: 10.1038/srep25659 (PMC4860631; doi:10.1038/srep25659)
Supplement: Supplementary Tables [file srep25659-s1.pdf]

Title: Pharmacokinetics and tolerability of NSC23925b, a novel P-glycoprotein inhibitor: preclinical study in mice and rats

Yan Gao[1,2], Jacson K. Shen[1], Edwin Choy[1], Zhan Zhang[3], Henry J. Mankin[1], Francis J. Hornicek[1], Zhenfeng Duan[1,*]

1 Sarcoma Biology Laboratory, Center for Sarcoma and Connective Tissue Oncology, Massachusetts General Hospital and Harvard Medical School, Boston, MA 02114

2 Department of Clinical Laboratory Diagnostics, Beijing Friendship Hospital, Capital Medical University, Beijing 100050, China

3 Shangqiu Medical College, Shangqiu 476100, Henan Province, China

* Corresponding author: Zhenfeng Duan, Sarcoma Biology Laboratory, Center for Sarcoma and Connective Tissue Oncology, Massachusetts General Hospital, 100 Blossom St, Jackson 1115, Boston, MA 02114, USA. Phone: 617-724-3144; Fax: 617-726-3883.

E-mail address: zduan@mgh.harvard.edu

Table S1. Plasma pharmacokinetic parameters of NSC23925b (Data provided as 95% confidence interval**)**

Pharmacokinetic Parameters	BALB/c Mice				SD Rats			
			I.V. 2.50 mg/kg				I.V. 2.50 mg/kg	
	I.P. 5.00 mg/kg	I.V. 2.50 mg/kg	Combined with Paclitaxel 5.00mg/kg	Combined with Doxorubicin 2.00mg/kg	I.P. 5.00 mg/kg	I.V. 2.50 mg/kg	Combined with Paclitaxel 5.00mg/kg	Combined wi[illegible] Doxorubici[illegible] 1.00mg/kg
K_{el} (h^{-1})	-0.008, 0.192	0.097, 0.183	0.115, 0.255	0.065, 0.163	0.013, 0.152	0.066, 0.091	0.059, 0.110	0.061, 0[illegible]
$t_{1/2}$ (h)*	-1.687, 19.067	3.369, 6.651	2.397, 5.223	3.725, 8.615	-0.119, 18.599	7.517, 10.203	5.664, 10.976	8.442, 11.0[illegible]
AUC_{0-t} ($h \cdot ng \cdot mL^{-1}$)	2874.927, 3853.073	1352.983, 1919.017	1435.570, 1812.430	1465.257, 1966.743	956.509, 1815.491	637.153, 894.847	603.352, 846.648	708.396, 1249.604
AUC_{0-inf} ($h \cdot ng \cdot mL^{-1}$)	1949.746, 6016.254	1378.261, 2023.739	1486.141, 1811.859	1643.784, 2010.216	128.628, 4116.628	759.498, 1044.502	645.591, 1028.409	860.813, 1521.187
$AUMC_{0-t}$ ($h \cdot h \cdot ng \cdot mL^{-1}$)	13387.664, 37270.336	6668.095, 13117.905	7898.075, 10345.925	9950.462, 11509.538	-601.903, 27257.903	4717.014, 6290.986	3684.984, 6535.016	5324.372, 9569.628
$AUMC_{0-inf}$ ($h \cdot h \cdot ng \cdot mL^{-1}$)	-38835.878, 140323.878	6629.740, 17260.260	9233.901, 10490.099	11696.442, 17103.558	-51954.103, 128854.103	8908.167, 12095.833	3395.906, 14930.094	10236.084[illegible] 20821.91[illegible]
CL ($mL \cdot kg^{-1} \cdot min^{-1}$)	/	19.734, 29.466	22.857, 27.743	20.471, 25.129	/	38.703, 53.897	38.308, 61.892	25.096, 45.504
MRT_{IV} (h)	/	4.915, 9.065	5.568, 6.392	5.944, 9.856	/	10.429, 12.971	6.208, 15.592	10.607, 15.3[illegible]
Vd_{SS} ($L \cdot kg^{-1}$)	/	8.294, 12.306	7.687, 10.493	7.225, 14.375	/	24.803, 39.997	22.792, 42.008	19.109, 36.0[illegible]
t_{max} (h)	-0.650, 3.650	/	/	/	-6.072, 10.412	/	/	/
C_{max} ($ng \cdot mL^{-1}$)	158.726, 437.274	/	/	/	18.021, 237.979	/	/	/
F (%)	57.368, 176.832	/	/	/	-7.076, 228.276	/	/	/

Abbreviations: AUC_{0-t}, area under the concentration-time curve calculated from zero up to the last measured concentration; AUC_{0-inf}, area under the concentration-time curve extrapolated from zero up to infinity; $AUMC_{0-t}$, area under the first moment of the concentration-time curve from zero up to the last measured concentration; $AUMC_{0-inf}$, area under the first moment of the concentration-time curve from zero up to infinity; CL, clearance; C_{max}, maximum plasma concentration; F, bioavailability; MRT_{IV}, mean residence time of a drug administered intravenously; I.P., intraperitoneal injection; I.V., intravenous injection; K_{el}, elimination rate constant; t_{max}, time to maximum observed concentration; $t_{1/2}$, elimination half life; Vdss, volume of distribution at steady-state. *$P < 0.05$

Table S2. Plasma pharmacokinetic parameters of paclitaxel and doxorubicin (Data provided as 95% confidence interval**)**

Pharmacokinetic Parameters	Paclitaxel				Doxorubicin			
	BALB/c Mice		SD Rats		BALB/c Mice		SD Rats	
	I.V. 5.00 mg/kg	Combined with NSC23925b 2.50mg/kg	I.V. 5.00 mg/kg	Combined with NSC23925b 2.50mg/kg	I.V. 2.00 mg/kg	Combined with NSC23925b 2.50mg/kg	I.V. 1.00 mg/kg	Combined [illegible] NSC23925[illegible] 2.50mg/k[illegible]
K_{el} (h^{-1})	0.282, 0.788	0.368, 0.538	0.050, 0.071	0.109,0.149†	0.023, 0.114	0.035, 0.075	0.016, 0.123	0.041,0.05[illegible]
$t_{1/2}$ (h)	0.635, 2.025	1.255, 1.825	9.671, 13.529	4.541, 6.259†	2.532, 18.868	8.058, 17.542	3.648, 17.352	12.140, 16.[illegible]
AUC_{0-t} ($h \cdot ng \cdot mL^{-1}$)	1913.140, 3094.860	1145.714, 3062.286	2978.558, 5421.442	4127.854, 7226.146	182.770, 231.230	85.260, 230.740	1.319, 178.081	92.190, 117.810
AUC_{0-inf} ($h \cdot ng \cdot mL^{-1}$)	1917.244, 3128.756	1188.127, 3079.873	3324.493, 5891.507	4255.093, 7278.907	138.684, 405.316	147.246, 288.754	36.315, 197.685	118.047, 159.953
$AUMC_{0-t}$ ($h \cdot h \cdot ng \cdot mL^{-1}$)	1844.192, 3611.808	1440.419, 3843.581	10090.503, 16371.497	11495.419, 13898.581	702.875, 2237.125	770.131, 1663.869	-254.095, 1146.095	577.898, 834.102
$AUMC_{0-inf}$ ($h \cdot h \cdot ng \cdot mL^{-1}$)	1917.958, 3904.042	1891.892, 4002.108	19353.337, 40430.663	15215.092, 15954.908†	-2032.137, 10346.137	1315.668, 6216.332	9.667, 2626.333	1659.518[illegible] 2806.482[illegible]
CL ($mL \cdot kg^{-1} \cdot min^{-1}$)	25.454, 40.946	23.762, 55.838	13.409, 22.991	10.802, 18.398	70.390, 181.610	107.334, 202.666	44.000, 258.000	101.207, 138.793
MRT_{IV} (h)	0.795, 1.525	1.184, 1.596	4.382, 8.618	1.965, 3.475†	0.424, 28.776	6.948, 27.652	5.117, 17.083	14.186, 18.014
Vd_{SS} ($L \cdot kg^{-1}$)	1.247, 3.373	1.642, 5.018	3.803, 10.457	1.121, 3.679†	55.844, 154.156	42.083, 279.917	48.689, 145.511	107.137, 124[illegible]

Abbreviations: AUC_{0-t}, area under the concentration-time curve calculated from zero up to the last measured concentration; AUC_{0-inf}, area under the concentration-time curve extrapolated from zero up to infinity; $AUMC_{0-t}$, area under the first moment of the concentration-time curve from zero up to the last measured concentration; $AUMC_{0-inf}$, area under the first moment of the concentration-time curve from zero up to infinity; CL, clearance; MRT_{IV}, mean residence time of a drug administered intravenously; I.P., intraperitoneal injection; I.V., intravenous injection; K_{el}, elimination rate constant; $t_{1/2}$, elimination half life; Vdss, volume of distribution at steady-state. †$P < 0.05$
